# Tumoral cavitation in colorectal cancer patients with unresectable lung metastasis treated with bevacizumab and chemotherapy

**DOI:** 10.1007/s00432-018-2656-y

**Published:** 2018-05-17

**Authors:** Yu Peng, Ye Chen, Xi Zhang, Yu Yang, Dan Cao, Feng Bi, Zhipi Li, Hongfeng Gou

**Affiliations:** 1 Department of Abdominal Cancer, The State Key Laboratory of Biotherapy, West China Medical School, Cancer Center, West China Hospital, Sichuan University, No. 37, Guo Xue Xiang, Chengdu, 610041 Sichuan China; 2 Department of Radiotherapy, The Affiliated Hospital of Hebei University, Baoding, China

**Keywords:** Colorectal cancer, Lung metastasis, Bevacizumab, Antiangiogenic agent, Cavitation

## Abstract

**Purpose:**

Efficacy and the frequency of tumor cavitation have not been investigated in patients with metastatic colorectal cancer (mCRC) and lung metastases (LMs) treated with chemotherapy in combination with bevacizumab. This study was aimed to evaluate the efficacy and safety of bevacizumab for unresectable mCRC with LM and to determine the frequency of tumor cavitation, and its correlation with clinical outcomes in patients receiving bevacizumab plus chemotherapy.

**Methods:**

Patients with mCRC and LMs treated with bevacizumab as first- or second-line therapy at West China Hospital, Sichuan University Cancer Center from September 2010 to November 2016 were included in this retrospective study. Data on clinicopathological characteristic were collected and overall survival (OS), progression-free survival (PFS), objective response rate (ORR) and disease control rate (DCR) were determined.

**Results:**

Among 60 patients included in the study, response rate (RR), stable disease (SD), and DCR were 43.6% (17/39), 51.3% (20/39) and 94.9% (37/39) in patients receiving bevacizumab as first-line treatment. Median OS and PFS of the first-line treatment group were 32.4 and 15.5 months, respectively. Among 60, 12 patients (20%) developed cavitation after bevacizumab therapy initiation. Median OS was longer in patients with cavitation than those without cavitation (42.1 vs 30.8 months; *p* = 0.042) in the first-line treatment group.

**Conclusion:**

Bevacizumab in combination with chemotherapy exhibited promising efficacy in mCRC patients LMs. Moreover, our findings reveal that OS might be affected by new tumor cavitation during antiangiogenic agent treatment.

## Introduction

Colorectal cancer (CRC) is one of the most common cancers worldwide, with an estimated 1.4 million new cancer cases and 693,900 deaths in 2012 (Torre et al. 2015). In 2013, there were over 348,000 new cases and 165,000 deaths due to CRC, which was thus the fourth most common cancer and the fifth most common cause of cancer-related deaths (Chen et al. 2017). Lung is the most common extra-abdominal site of metastasis from CRC, with pulmonary metastasis rates ranging from 10 to 25% in patients with CRC (Iwasaki et al. 2005; Inoue et al. 2004; Mitry et al. 2010; Penna et al. 2002). One-third of these patients had isolated lung metastases (LMs), whereas the remaining two-thirds had accompanying metastases in other locations, particularly the liver. The prognosis of CRC patients with metachronous metastases or LMs alone were better than those of CRC patients with synchronous metastases or metastases in other locations (Mitry et al. 2010). Although surgical resection is the best choice for patients with LMs, only 4.1% of synchronous LMs and 14.8% of metachronous LMs were completely resected (Mitry et al. 2010). For patients with unresectable LMs, chemotherapy combined with targeted agents is the treatment of choice.

Bevacizumab (BV), a monoclonal antibody directed against vascular endothelial growth factor. A inhibits the formation of new blood vessels supplying the tumors and normalizes tumor vasculature (Presta et al. 1997). Bevacizumab was shown to improve response rates (RRs), overall survival (OS) and progression-free survival (PFS) when used in combination with standard chemotherapy treatments in patients with metastatic CRC (mCRC) (Fuchs et al. 2007; Giantonio et al. 2007; Saltz et al. 2008). Bevacizumab combined with chemotherapy regimen was also shown to improve survival in patients with non-small cell lung cancer. Cavitation formation is a typical radiological phenomenon reported in cancer patients receiving antiangiogenic therapy for lung lesion. Cavitary changes encompass new appearance of a visible air-filled cavity in a solid LM and an increase in cavity size with a decrease in the solid component of a pre-existing cavitary LM following treatment (Lim et al. 2015). Tumor cavity formation reported to occur in 14–24% of patients with primary lung cancer treated with anti-angiogenic therapy (Crabb et al. 2009; Marom et al. 2008; Nishino et al. 2012). However, among patients with primary lung cancer, no statistically clinical differences in PFS and OS were found between patients who developed cavitation and those who did have cavitary lesions during therapy (Crabb et al. 2009; Marom et al. 2008; Nishino et al. 2012). Tumor cavitation of LM was observed in CRC patients treated with the multi-kinase inhibitor regorafenib (Ricotta et al. 2017; Lim et al.2015). In post hoc analysis of the phase III CORRECT trial, Riccardo et al. found that 34.3% (24/70) of the patients had *de novo* cavitation among those with LMs who were treated with regorafenib. While LM cavitation represents a novel radiological marker of favorable outcome, whether tumor cavitation in CRC patients with LMs treated with bevacizumab has not been reported. The purpose of the present study was to evaluate the efficacy of bevacizumab for advanced CRC with LM and to determine the frequency of tumor cavitation, and its correlation with clinical outcomes in patients receiving bevacizumab in combination with chemotherapy.

## Patients and methods

### Patients

In this retrospective study, patients with histologically proven CRC and evidence of LM, who were treated with bevacizumab and chemotherapy from September 2010 to November 2016, at the West China Hospital, Sichuan University Cancer Center, were identified from the medical database. LM diagnosis was based on chest computed tomography (CT) imaging. All patients with surgically unresectable metastases and those with ablatable or extra LMs were included.

### Methods

Bevacizumab was added to the palliative regimens including oxaliplatin-based, irinotecan-based regimens and so on in first or second line regimens.

XELOX-Bev treatment consisted of 90-min I.V. infusion of bevacizumab (7.5 mg/kg) on day 1, followed by oxaliplatin 130 mg/m2-i.v. infusion over 2 h on day 1 in combination with capecitabine orally at a dose of 2,000 mg/m^2^/day with first dose on the morning of day 1 and last dose on the evening of day 14 every 3 weeks. FOLFIRI-Bev treatment consisted of a 90-min I.V. infusion of bevacizumab (5 mg/kg) on day 1, followed by a 90-min I.V. infusion of irinotecan (180 mg/m^2^) on day 1, leucovorin (400 mg/m^2^) 2 h infusion on day 1, bolus fluorouracil (400 mg/m^2^) on day 1, 46 h infusion of fluorouracil (2400 mg/m^2^). FOLFOX-Bev treatment consisted of a 90-min I.V. infusion of bevacizumab (5 mg/kg) on day 1, followed by a 90-min I.V. infusion of oxaliplatin (130 mg/m^2^) on day 1, leucovorin (400 mg/m^2^) 2 h infusion on day 1, bolus fluorouracil (400 mg/m^2^) on day 1, 46 h infusion of fluorouracil (2400 mg/m^2^).

### Clinical evaluation and the follow-up

Before treatment initiation, patients were evaluated including the following: collection of medical history, physical examination, evaluation of Eastern Cooperative Oncology Group (ECOG) performance status (PS) score, full hematological tests, blood biochemistry (bilirubin, aspartate aminotransferase, alanine aminotransferase, albumin, serum lactate dehydrogenase, urea, creatinine, glucose, and serum electrolytes), and chest and full abdomen contrast spiral CT. Cavitation was defined as the presence of an air-filled cavity that is ≥ 10% of the maximum diameter of one or more LMs measuring ≥ 10 mm on CT scans. Radiological imaging markers predicting clinical outcomes in patients with mCRC treated with regorafenib: post hoc analysis of the phase III CORRECT trial (RadioCORRECT study).

Adverse effects, drug usage and other medical events were recorded at each visits. Hematological and biochemical tests were also performed. Following treatment completing, patients were followed up once every 3 months until death or loss to follow-up. The last follow-up was conducted on June 16th, 2017 by telephone interview or review of the medical records.

### Evaluation of treatment response, toxicity and survival

OS was calculated from diagnosis to death or the date of last follow-up (June 16th, 2017). PFS was defined as the period from initial treatment with bevacizumab to confirmation of progression by imaging examination according to the RECIST 1.1 criteria and disease control rate (DCR) was defined as the proportion of patients who achieved complete response, partial response (PR) or SD status. Treatment was continued until significant toxicity was observed or progressive disease (PD) was confirmed. Response evaluation was based on the RECIST criteria every 2–3 cycles for XELOX–bevacizumab regimen and 4–6 cycles for the FOLFIRI–bevacizumab regimen and FOLFOX–bevacizumab regimens. Tumor progression was evaluated based on with clinical assessment imaging results and tumor markers. Toxicity was evaluated according to the Common Terminology Criteria for Adverse Events v3.0.

### Statistical analysis

Association between two categorical variables, such as that between the presence of cavitation and chemotherapy regimen was evaluated by the Chi-square test or Fisher’s exact test as appropriate. The Kaplan–Meier method and log rank test were used for evaluation of the prognostic effect of each variable on OS and PFS, and Kaplan–Meier plots were shown to present difference in survival times. All statistical analyses were performed using the SPSS Statistic 18.0 software package. *p* values < 0.05 were considered to present significant difference among groups.

### Human rights

The study has been approved by the Ethics Committee of our hospital and has obtained an informed consent of patients and their families.

## Results

### Patient characteristics

Between April 2008 and November 2016, 60 patients mCRC with LMs were treated with bevacizumab plus chemotherapy. Demographic and clinical characteristics of the cohort are presented in Table 1. Briefly, there were 37 males and 23 females, and median age was 61 years (range 25–81 years). 53 and 7 patients had PS scores of 0 and 1, respectively. Primary tumor locations were rectum, left hemicolon and right hemicolon in 39, 15 and 6 cases, respectively. The primary tumor was resected in 47 cases (78.3%). Among a total of 60 patients, there were 43 cases and 17 cases with synchronous and metachronous metastases, respectively. The number of metastases over 5 were 46 cases (76.7%). In the entire study cohort, 18 cases (30%) patients had only LMs, whereas lung and liver metastasis were found in 34 cases (56.7%). Furthermore, 8 patients (13.3%) had lung, liver, and abdominal lymph node metastases. Finally, among 35 patients (58.3%) whose tumor KRAS status was determined, 10 patients (16.7%) had tumors with wild-type KRAS, whereas the tumors in 25 (40%) had mutant KRAS. None of the cases were lost to follow-up by June 16, 2017; therefore, the follow‑up rate was 100%. 26 patients had been dead in the last follow-up.

**Table 1:** Patient characteristics

Characteristics	Patients (*n*)	%
Gender		
Male	37	61.7
Female	23	38.3
Baseline ECOG PS		
0	53	88.3
1	7	11.7
Primary tumor		
Rectum	39	65
Left hemicolon	15	25
Right hemicolon	6	10
Primary tumor resection		
No	13	21.7
Yes	47	78.3
Timing of metastases		
Synchronous	43	71.7
Metachronous	17	28.3
Number of metastases		
≤5	14	23.3
>5	46	76.7
Lung metastasis only		
No	42	70
Yes	18	30

### Therapeutic regimens

Of a total of 60 patients, 39 underwent first-line chemotherapy in combination with bevacizumab (the first-line treatment group), whereas the remaining 21 patients underwent second-line chemotherapy including combination with bevacizumab (the second-line treatment group). In the first-line treatment group, there were 26 (66.7%) and 13 (33.3%) patients treated with oxaliplatin-based (FOLFOX and XELOX) and irinotecan-based (FOLFIRI) chemotherapeutic regimens, respectively.

Patients in the first-line treatment group received a median 7 (range 3–19) cycles of bevacizumab. Among those in the second-line treatment group, 11 (52.4%) and 10 (47.6%) patients were treated with oxaliplatin-based and irinotecan-based chemotherapy, respectively, with a median 6 (range 3–12) cycles of bevacizumab.

### Efficacy

**Fig. 1:**
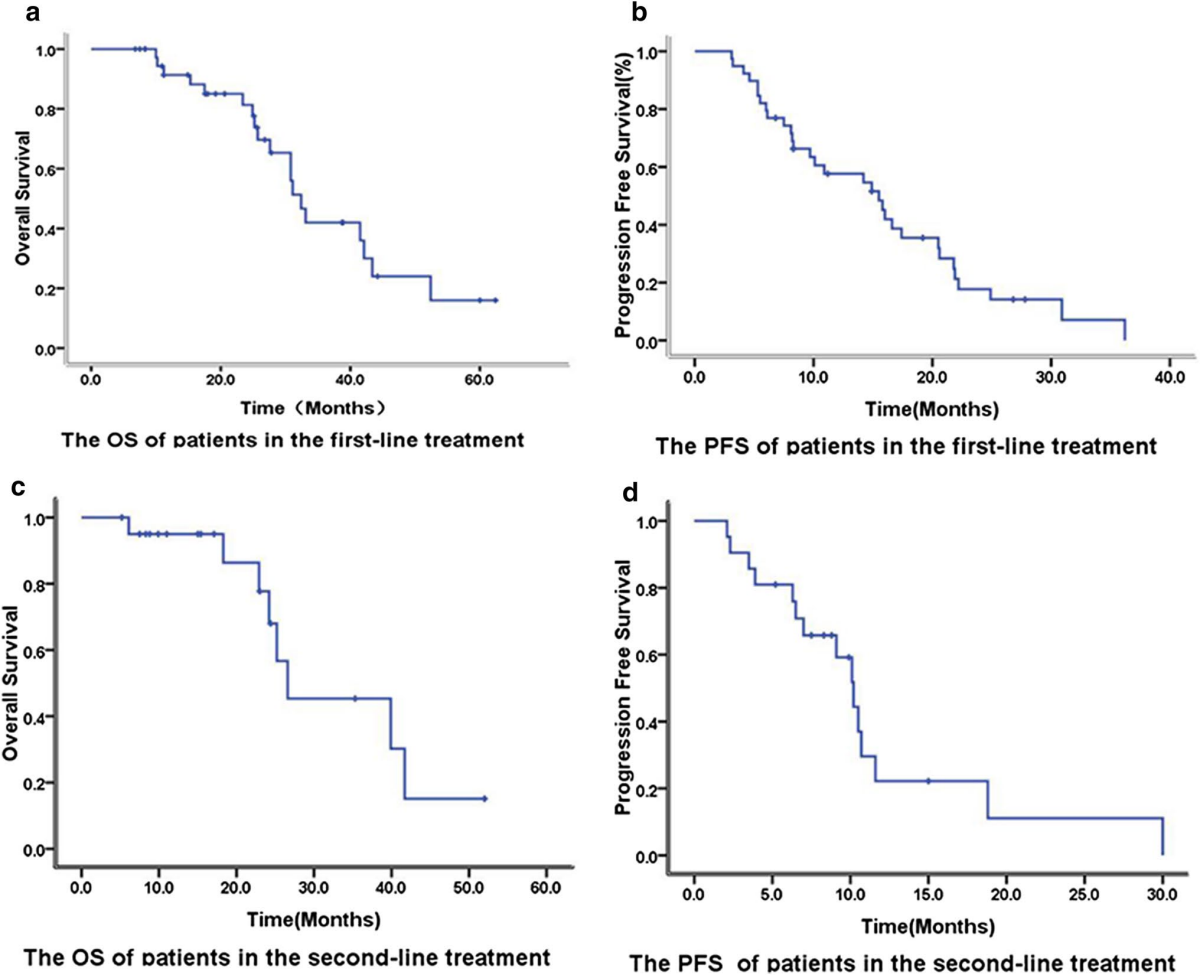
Overall survival (**a**) and Progression-free survival (**b**) of the first-line treatment. Overall survival (**c**) and Progression-free survival (**d**) of the second-line treatment

Median follow-up duration for the entire cohort was 22.7 months (range 6.1–60.0 months). RR, SD, and DCR rates were 43.6% (17/39), 51.3% (20/39), and 94.9% (37/39), respectively, in the first-line treatment group. In addition, the median OS and PFS in the first-line treatment group were 32.4 and 15.5 months, respectively (Fig. 1). There were only 14 patients with LMs in the first-line treatment group. Among the patients with LMs in the first-line treatment group, RR, SD, and DCR rates were 64.3% (9/14), SD 28.6% (4/14), and 92.9% (13/14), respectively, with a median PFS of 16.6 months. The OS and PFS of patients with LMs treated with bevacizumab as first-line therapy were not significantly and independently associated with age, baseline ECOG PS, primary tumor site, time of metastasis (synchronous or metachronous), primary tumor resection (yes or no), number of metastases (≤ 5 or > 5), lung metastasis only or presence of metastasis in other locations.

In the second-line treatment group, RR, SD, and DCR were 19.0% (4/21), 66.7% (14/21) and 85.7% (18/21), respectively. Median OS and PFS were 26.6 and 10.2 months, respectively, in the second-line treatment group (Fig. 1). There were only four patients with LMs who received bevacizumab as second-line treatment. Among these patients with LMs in the second-line treatment group, PR, SD, and PD were observed in 1, 2 and 1 patients, respectively.

**Fig. 2:**
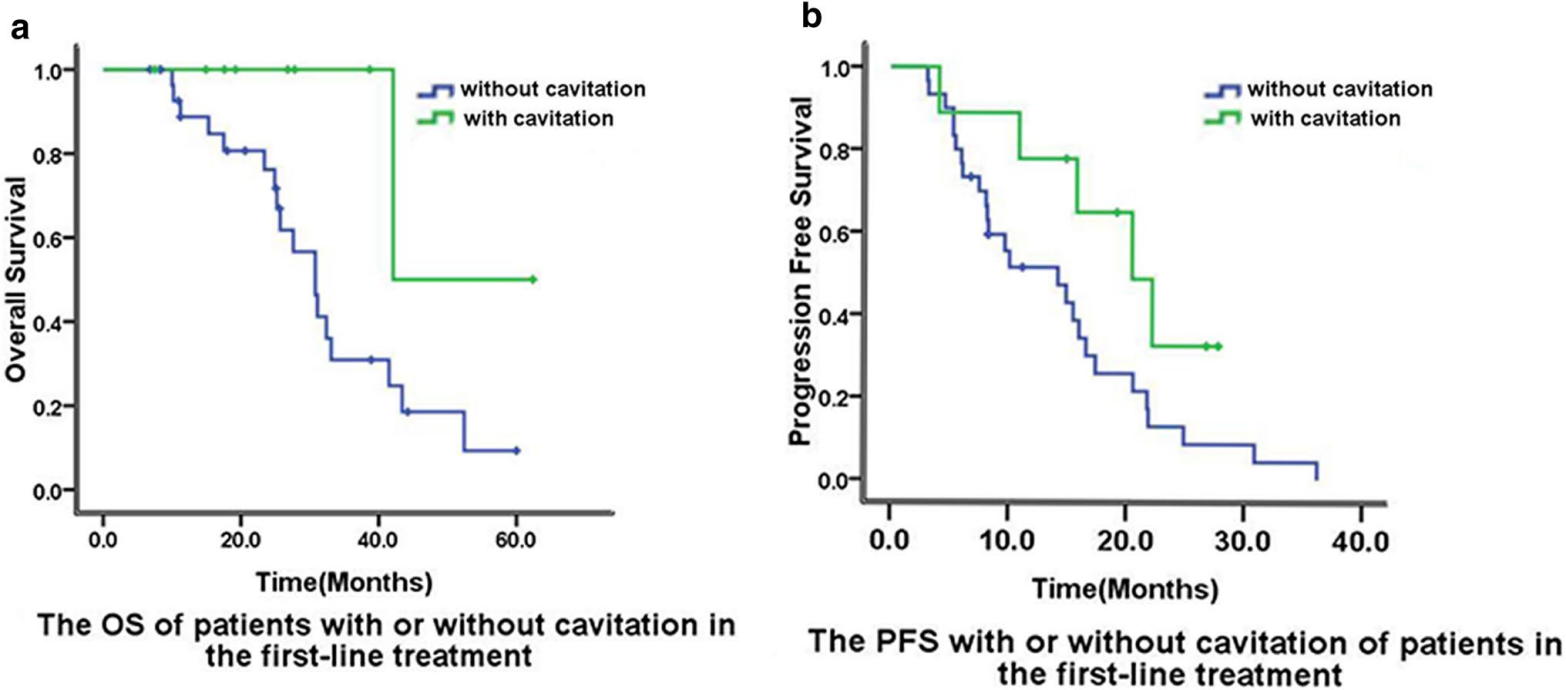
Overall survival (**a**) and progression-free survival (**b**) were compared between patients who developed cavitation vs those who did not in the first-line treatment

**Fig. 3:**
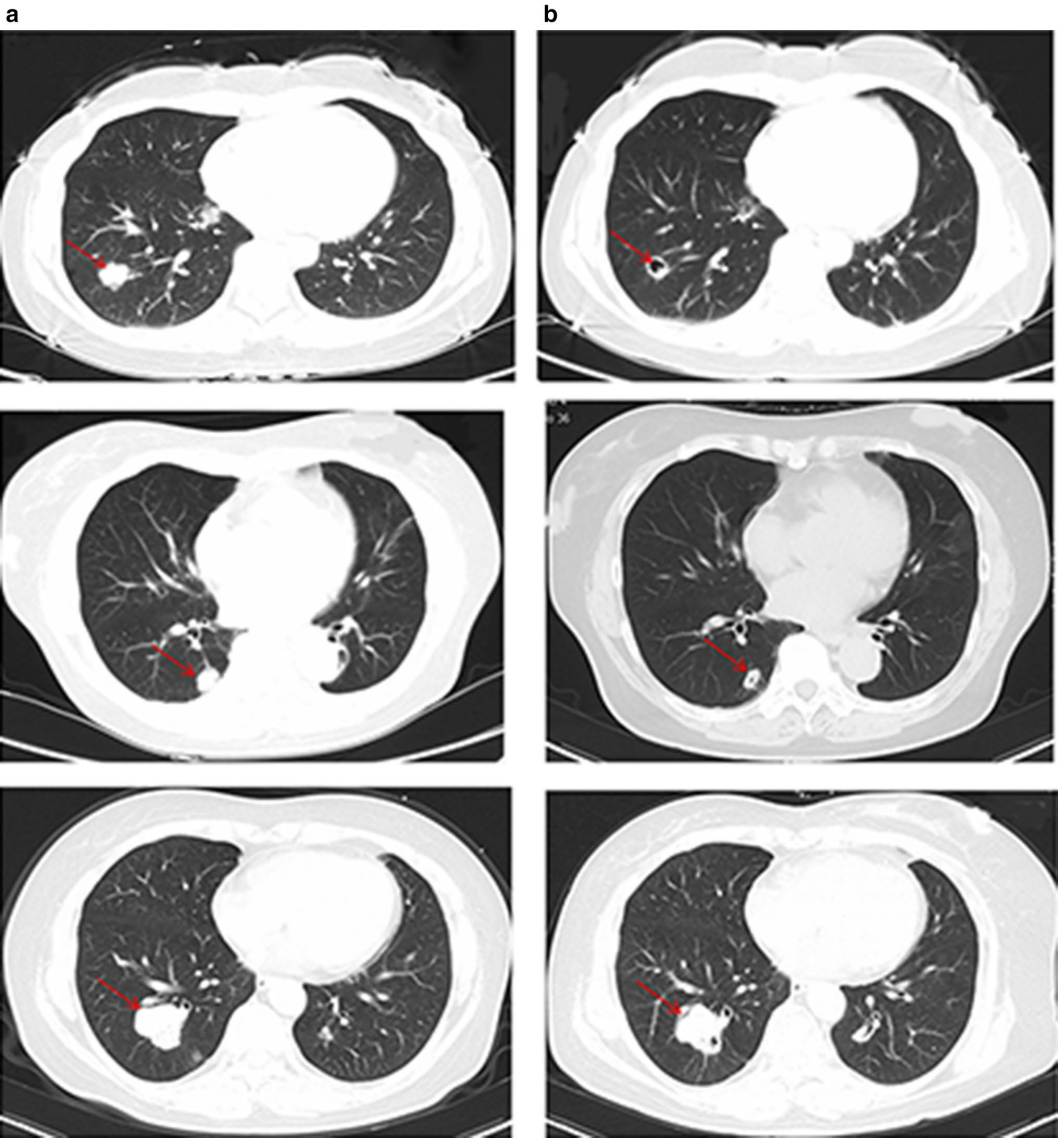
Baseline (**a**) and after therapy (**b**) CT displaying the onset of cavitation in three patients treated with bevacizumab. The arrow highlights tumor metastases with cavitation

Out of 60 patients, 12 patients (20%) developed cavitation after the initiation of bevacizumab therapy. Cavitation was found in the patients who achieved PR and SD. Additionally, nine and three patients developed cavitation during the first-line treatment and second-line treatments, respectively.We showed the typical images of lung cavitation in three patients in Fig. 3. Cavitation formation in lung lesions after the initiation of bevacizumab therapy was not associated with sex, primary tumor location, number of metastases, or chemotherapy regimens. Median OS of patients with cavitation was longer than that of patients without cavitation (42.1 vs 30.8 months, *p* = 0.042) in the first-line treatment group (Fig. 2). Median PFS of patients with and without cavitation were 20.5 months and 14.2 months, respectively, in the first-line treatment (*p* = 0.07) (Fig. 2). The median PFS (*p* = 0.19) and OS (*p* = 0.25) of patients with CRC and LMs treated with bevacizumab as second-line therapy were not significantly and independently associated with cavitation formation.

### Toxicity

All 60 patients were evaluable for toxicity (Table 2). The incidence of grade 3/4 leukopenia, neutropenia, anemia and thrombocytopenia was 1.7, 8.3, 3.3 and 6.7% respectively. Other grade 3/4 adverse event was nausea/vomiting (1.7%). Grade 3 hypertension was reported in one patient. No.

**Table 2:** Toxicity

	Number of toxicity (%)	
	Grade 1–2	Grade 3–4
Leukopenia	35 (58.3)	1 (1.7)
Neutropenia	27 (45)	5 (8.3)
Anemia	43 (71.1)	2 (3.3)
Thrombocytopenia	28 (46.7)	4 (6.7)
Hand and foot syndrome	9 (15)	0 (0)
Nausea/vomiting	18 (30)	1 (1.7)
Diarrhea	10 (16.7)	0 (0)
Allergic reaction	5 (8.3)	0 (0)
Hypertension	12 (20)	1 (1.7)
Venous thrombosis	1 (1.7)	0 (0)
Proteinuria	4 (6.7)	0 (0)

Grade 3/4 allergic reaction, diarrhea, proteinuria was observed. No hemoptysis was reported in all of the 60 patients. There was no treatment-related death.

## Discussion

This retrospective study demonstrated that bevacizumab in combination with standard chemotherapy regimens as a first- or second-line option might be associated with better outcomes as a first-line and second-line option in patients with mCRC and LMs. Additionally, our analysis revealed that 20% (12/60) of the patients with mCRC and LMs developed cavitary lesions in their lungs during bevacizumab including treatment. To our knowledge, this is the first study that focused on the efficacy of bevacizumab for LM in mCRC patients and the first to report the rate of tumor cavitation associated with bevacizumab therapy in these patients.

Addition of bevacizumab to standard chemotherapy regimens resulted in PFS ranging between 7.3 and 12.8 months, OS ranging between 12.9 and 49.9 months, and RR ranging between 19 and 47% (Fuchs et al. 2007; Giantonio et al. 2007; Saltz et al. 2008; Guan et al. 2011; Ilic et al. 2016; Yin et al. 2016). The current study showed that OS, PFS and RR were better after the addition of bevacizumab as first- or second-line treatment. The median OS and PFS with bevacizumab as first-line treatment were 32.4 and 15.5 months, respectively, whereas those with bevacizumab as second-line treatment were 26.6 and 10.2 months, respectively; these results were comparable to those of previous studies (Fuchs et al. 2007; Giantonio et al. 2007; Saltz et al. 2008; Guan et al. 2011; Ilic et al. 2016; Yin et al. 2016). We further determined that the efficacy of bevacizumab in combination with standard chemotherapy regimens in mCRC patients with LMs was comparable to that in mCRC patients with non-lung metastasis. Our findings also showed that the toxicity was tolerable and similar to that reported by other studies.

Tumor cavitation induced by antiangiogenic agents is frequently observed in non-small cell lung cancer patients. Regorafenib is an oral multikinase inhibitor that targets a broad range of angiogenic, stromal, and oncogenic kinases, which has been approved for salvage therapy of advanced CRC (Wilhelm et al. 2011; Grothey et al. 2013). In the current study, we also observed tumor cavitation of LMs in patients with mCRC treated with regorafenib. The post analysis of the phase III CORRECT trial, Riccardo et al. found that 34.3% (24/70) patients had denovo cavitation among those with LMs treated with regorafenib (Ricotta et al. 2017). Furthermore, Lim et al. also reported that 17 patients (32.1%) of a cohort of 53 CRC patients with LMs developed tumor cavitation during regorafenib treatment (Lim et al. 2015). Other antiangiogenic agents including sorafenib, sunitinib, and imatinib were also shown to provoke necrosis and cavitations by reducing tumor vascularization in renal cell carcinoma, gastrointestinal stromal tumors, and hepatocellular cancer (Abou-Alfa et al. 2006; Motzer et al. 2006; Benjamin et al. 2007; Choi et al. 2007; Horger et al. 2009; Le Cesne et al. 2009).

The present study is also the first to report the frequency of cavitation in LM of CRC during bevacizumab therapy. Cavitation development observed in 20% (12/60) of the study patients was similar to that reported in primary lung cancer patients treated with bevacizumab. Bevacizumab was shown to be associated with organ perforation (e.g., bowel or nasal) in patients with several solid malignancies (Traina et al. 2006; Gray et al. 2007). The exact pathologic mechanism leading to organ perforation is not clear. Tumor cavitation, which may be considered to involve a mechanism similar to that underlies, perforation, might result from central tumor necrosis induced by the inhibition of tumor-associated angiogenesis, as shown in preclinical models (Presta et al. 1997; Johnson et al. 2004; Laurie et al. 2006).

The correlation between cavitation and prognosis remains controversial. In the current study, we found that OS was different between patients with and without cavitation. The post analysis by Riccardo et al. also found that LM cavitation represents a novel radiological marker of favourable outcomes in the CORRECT III trial (Ricotta et al. 2017). Choi et al. reported that a reduction of 15% in tumor density correlated with PFS than RECIST in patients with metastatic gastrointestinal stromal tumor treated with imatinib mesylate (Choi et al. 2007). Similar imaging changes not only occur in lung lesions but also in liver metastatic lesions in mCRC patients treated with bevacizumab. However, the rate of such changes in liver is observed in less than 5% of these patients, and the liver lesions include fibrosis and necrosis. Choi et al. also reported that the morphological changes were significantly associated with pathological response and OS in liver metastasis of CRC (Choi et al. 2007). In contrast, several studies on primary lung cancer failed to find significant clinical differences in outcomes such as PFS and OS between patients with or without cavitation due to therapy (Crabb et al. 2009; Marom et al. 2008; Nishino et al. 2012). Furthermore, there was no correlation between cavitation and treatment outcomes in CRC patients treated with regorafenib (Lim et al. 2015). Further studies should investigate the impact of cavitation on prognosis.

The current study has several limitations including the retrospective design, the small number of patients from a single institution, and the relatively short follow-up period. Detailed radiological patterns of LMs with cavitation were not investigated due to the limited number of patients. Given that the frequency of tumor cavitation was 25%, studies including larger cohorts will be necessary to further investigate differences in clinical and survival data across patient groups with different cavitation patterns.

In conclusion, the current retrospective study revealed that tumor cavitation was found in 20% of CRC patients with LMs who were treated with bevacizumab therapy. Furthermore, the prognosis might be better in patients with cavitation compared to those without cavitation. However, further investigation is necessary to verify the association between tumor cavitation and prognosis in patients with CRC and LMs.
